# Schwann Cells in the Tumor Microenvironment: Need More Attention

**DOI:** 10.1155/2022/1058667

**Published:** 2022-02-10

**Authors:** Leqi Sun, Shuhai Chen, Mingyou Chen

**Affiliations:** ^1^Department of Oncological Medical Services, Graduate School of Biomedical Sciences, Tokushima University, Tokushima 770-8503, Japan; ^2^Department of Digestive and Transplant Surgery, Graduate School of Biomedical Sciences, Tokushima University, Tokushima 770-8503, Japan; ^3^Department of Cardiology, The First Affiliated Hospital of Shandong First Medical University & Shandong Provincial Qianfoshan Hospital, Shandong Medicine and Health Key Laboratory of Cardiac Electrophysiology and Arrhythmia, Jinan, Shandong 250014, China

## Abstract

The tumor microenvironment (TME), which is composed of various cell components and signaling molecules, plays an important role in the occurrence and progression of tumors and has become the central issue of current cancer research. In recent years, as a part of the TME, the peripheral nervous system (PNS) has attracted increasing attention. Moreover, emerging evidence shows that Schwann cells (SCs), which are the most important glial cells in the PNS, are not simply spectators in the TME. In this review article, we focused on the up-to-date research progress on SCs in the TME and introduced our point of view. In detail, we described that under two main tumor-nerve interaction patterns, perineural invasion (PNI) and tumor innervation, SCs were reprogrammed and acted as important participants. We also investigated the newest mechanisms between the interactions of SCs and tumor cells. In addition, SCs can have profound impacts on other cellular components in the TME, such as immune cells and cancer-associated fibroblasts (CAFs), involving immune regulation, tumor-related pain, and nerve remodeling. Overall, these innovative statements can expand the scope of the TME, help fully understand the significant role of SCs in the tumor-nerve-immune axis, and propose enlightenments to innovate antitumor therapeutic methods and future research.

## 1. Introduction

In recent decades, cancer research has exponentially expanded beyond the topic of cancer cells alone to include complex and extensive heterotypic interactions between cancer cells and the tumor microenvironment (TME) [1]. Therefore, intensive discussions have been made among the components of the TME, including cells (e.g., stromal cells and immune cells), vasculature systems (blood vessels and lymphatic vessels), widely existing extracellular matrix (ECM), and networks constituted by various signaling molecules [2–4]. Many studies have expounded their supporting role in many important biological behaviors of cancer, such as metabolic support [5], immunosuppression [6], angiogenesis [7], invasion and metastasis [8], and chemotherapy resistance [9]. Similar to the vascular system, the nervous system, which is distributed throughout all organs of the body, can also be an important pathological factor in the TME [10, 11]. Much emerging evidence has confirmed the crosstalk between cancer and the peripheral nervous system (PNS) [12, 13]. This link is usually related to the adverse outcomes of tumors and has become the hotspot of current research [14–16]. However, for a long time, studies have mainly focused on the carcinogenic effects of neurons in the PNS [17, 18], which leads to an ignorance of glial cells. Moreover, from the therapeutic perspective, although targeting nerve fibers in tumors can inhibit tumor growth and metastasis [17, 19–21], the neuronal and non-neuronal toxicity caused by this method hinders its clinical application. Schwann cells (SCs) are the most important type of glial cells in the PNS and exist in almost every anatomical part of the body [22, 23]. Their high plasticity and absolute abundance enable them to recruit several immune cells, regulate the microenvironment, and assist regeneration [24, 25]. These factors make them perfect candidates hijacked by tumor cells to form and maintain a unique TME [26–28]. Some groundbreaking articles focused on the characteristics of SCs in the TME and their supporting effects on cancer, which adds an exciting new dimension to the interaction between tumors and the TME [28–33]. Nevertheless, compared to a great number of studies on glial cells of the central nervous system in the background of breast cancer or lung cancer brain metastasis [34, 35], SCs did not attract sufficient attention in the context of cancer. Thus, we focused on the newest progress in the study of SCs in non-neuronal cancers and innovatively put forward our points of view. In particular, in the niche formed by tumor-nerve interactions in the process of cancer occurrence and development, what changes have occurred in SCs and what role will they play? What are the direct effects of SCs on tumor cells? In addition to tumor cells, which cell components in the TME can SCs interact with and what molecular mechanism is under it? The answers to these questions will help one comprehensively understand the role of nerves and their cellular contents in cancer progression, further expand the scope of the TME, and propose directions to innovate antitumor strategies and follow-up research.

## 2. SCs in the “Tumor-Nerve Niche”

The peripheral nerve sheath is composed of three layers of connective tissues from the outside to the inside, i.e., the epineurium, perineurium, and endoneurium. As the main support cells of PNS, SCs constitute 90% of the endoneurial space [23]. In addition, SCs are present near neurites or nerve endings and exist in almost every anatomical part of the body [22]. The presence of SCs has been observed in precancerous tissues of pancreatic cancer and colon cancer [36], which indicates that SCs may be involved in the formation of the TME and precancerous niche in various tissues and become an early mark and driving factor in the early stages of tumor development [30, 37]. When perineural invasion (PNI) occurs in tumors, SCs are very important participants. In the microenvironment where tumors cause nerve damage, SCs can obtain repair phenotypes and functions similar to those in the process of nerve regeneration through adaptive reprogramming, which is closely related to increased innervation in cancer [23, 28, 32, 38, 39]. Therefore, in the scope of two main patterns between tumor-nerve interactions, PNI, and tumor innervation [40], we illustrate the changes and roles of SCs in this special ecological niche.

### 2.1. SCs and PNI

Due to the close correlation with the recurrence, metastasis, and poor prognosis of many types of cancers, PNI has been considered a potential pathway for distant spread of tumor cells, similar to hematogenous metastasis and lymphatic metastasis [38, 41]. Originally, nerves were only considered the mechanical passageway for cancer transmission, and PNI was defined as tumor cells that invade any of the three layers of the neurilemma structure [42]. Recently, in the study of head and neck cancer, Bakst et al. proposed an emerging consensus that defined the process of cancer cells invading the outer surfaces of nerves as periodic tumor spread and their invasion into the interior as PNI [43]. We consider that this conceptual update helps to emphasize the role of SCs in cancers due to their position in the endoneurial space. *In vitro* studies reveal that through the interaction of chemokines and their receptors, SCs achieve a strong affinity for tumor cells instead of benign cells. Then, through early physical contact with SCs, tumor cells are dispersed, marked, and finally recruited into the nerve [28, 33, 36]. When tumor cells invade the endoneurium and damage axons, they further trigger the cascade reaction of inflammatory cytokines to form a unique cellular and biochemical microenvironment around the nerve. In this microenvironment, the complex crosstalk among cells, including SCs and soluble factors, initiates a continuous and multistep process, PNI, which promotes tumor progression [38, 41, 44, 45].

### 2.2. Reprogramming of SCs

Dyachuk et al. revealed the presence of Schwann cell precursors (SCPs), which have a similar transcription spectrum to SCs and neural crest cells [46]. These SCPs can differentiate into abnormally diverse cell types, including immature SCs, and subsequently into myelinated and unmyelinated SCs. This ability implies the plastic potential of SCs, which makes them a pluripotent cell pool to develop and regenerate PNS [47]. In fact, SCs are eminent in their ability to naturally (adaptively) reprogram and reprogram the surrounding environment [26]. After peripheral nerve injury, myelinated SCs dedifferentiate into “repair SCs” (rSCs) with an unmyelinated phenotype, change the local signal environment through matrix remodeling and release of proinflammatory mediators, recruit macrophages to cooperatively eliminate the myelin fragments, and guide the axon genesis, thus carving out the way for subsequent nerve regeneration [48–51]. This process of SC activation and transdifferentiation into a repair phenotype in nerve injury is a typical representative of natural (adaptive) reprogramming [26]. Transcriptomics revealed that genes encoding structural proteins such as myelin transcription factor Egr2, myelin protein zero, and myelin basic protein were downregulated. Meanwhile, proteins expressed in rSCs were re-expressed, such as glial fibrous acid protein (GFAP) and p75 neurotrophin receptor (p75NTR). Simultaneously, rSCs release more chemokines and neurotrophic factors [24, 48, 52]. In addition to the nerve, new research results show that injury in other tissues can activate the repair program of SCs and promote repair and regeneration [53, 54]. Parfejevs et al. described a similar phenotypic transition of SCs in the context of skin wound healing, where rSCs could spread from injured nerves to granulation tissue and support non-neural tissue repair [55]. In multiple types of cancer, such as thyroid cancer, salivary duct carcinoma, cutaneous squamous cell carcinoma, pancreatic cancer, and colon cancer, when the nerve is damaged or invaded by cancer cells, the number of GFAP-positive SCs increases [33, 36]. Silva et al. used bioinformatic analysis to investigate the transcriptomics of SCs among lung cancer samples and found that dedifferentiation-related pathways were overexpressed [56]. In cutaneous melanoma, the local neurodegenerative response caused by cancer is very similar to the nerve repair process induced by skin injury. The genetic, transcriptional, and functional phenotypes are similar between reprogrammed SCs in tumors and rSCs induced by nerve injury. These reprogrammed SCs had stronger moveability, increased secretion of neurotrophic factors, attracted macrophages, and polarized them into the M2 type, which accelerated the tumor growth and formation of metastasis in vivo [32, 52]. Recently, Shurin et al. paid attention to the fact that surgical intervention might potentially promote the local and distant recurrence of cancers and the involved cancer-SC-nerve axis. It is speculated that stress reactions and wound healing processes caused by surgery may also stimulate the tumor-related reprogramming of SCs and ultimately more strongly promote tumor metastasis [31].

In general, nerve damage caused by tumor invasion forms a unique microenvironment for the repair and regeneration of PNS. This process spontaneously reminds us of the concept of “cancer is a wound that will never heal” [57] and further enlightens us to explore the potential significance of SC regeneration-promoting characteristics in the “wound.”

### 2.3. SCs and Tumor Innervation

In addition to PNI, another important interaction pattern between cancer and nerves is “cancer-related neurogenesis,” which is also known as “tumor innervation” and is a process where cancer cells activate nerve-dependent pathways to recruit peripheral nerves into tumor bodies [10, 58, 59]. Indeed, solid tumors are physically innervated, and nerves are actively involved in tumor progression. An increase in nerve fiber density in tumors is associated with an increase in pathological stages [10–12, 27, 60, 61]. Hutchings et al. summarized the occurrence of tumor innervation and its pathophysiological features in prostate cancer, gastric cancer, colon cancer, lung cancer, and pancreatic cancer, i.e., five types of cancer that were widely discussed in this theme [62]. Recent studies have confirmed that the increase in nerve density in papillary thyroid carcinoma is positively correlated with extrathyroidal infiltration [63]. Innervation has also been identified as a feature in esophageal cancer and may be driven by nerve growth factor (NGF) released from tumor cells [64]. The density of nerve fibers in the TME has been recognized as an important new prognostic biomarker for patients with perihilar cholangiocarcinoma (pCCA). Combined with lymph node status, it can be used to perform risk stratification for poor postoperative results in pCCA patients [16]. Therefore, it has been proposed to list this tumor innervation phenomenon, which leads to increased tumor invasiveness, as a new hallmark of cancer [65]. Mechanistically, the above text proposed that SCs in the tumor-nerve niche were reprogrammed and released many neurotrophic factors and guiding factors. Therefore, there is reason to believe that reprogrammed SCs can promote axon genesis and extend into the tumor body via their functions in the nerve regeneration process, which contributed to tumor innervation [10, 18, 32]. This increased neurogenesis is accompanied by an increase in the production of neurotransmitters that facilitate the proliferation and spread of cancer, which ultimately further promotes tumor development [10, 66]. In addition, these neurotrophic factors secreted by SCs in the above process are the key molecules that promote PNI [33, 38, 41]. Simultaneously, considering the special position in the endoneurium of SCs, it is reasonable for SCs to further boost PNI.

**Figure 1:**
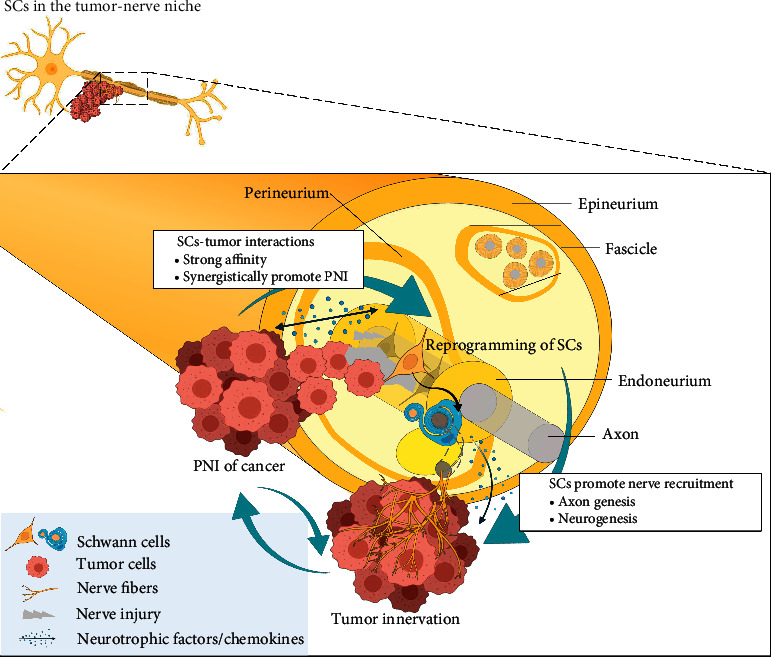
Schematic diagram of how Schwann cells (SCs) undergo reprogramming and facilitate perineural invasion (PNI) and tumor innervation. SCs show a strong affinity with cancer cells, even in the early stage of tumorigenesis. Tumors attracted by SCs (nerve) or developed actively invade the nerves to form the tumor-nerve niche, and SCs in this microenvironment undergo reprogramming. Reprogrammed SCs further promote nerve recruitment through axon genesis and follow neurogenesis, which finally induce the tumor innervation. Reprogrammed SCs can also synergistically promote PNI, thus constituting positive feedback of cancer-nerve crosstalk. Abbreviation: PNI, perineural invasion; SCs, Schwann cells.

By summarizing the above contents, we can draw an inference. In the early stage of tumorigenesis and tumor development, SCs can show a strong affinity to cancer cells. Tumors that attract, develop, and actively invade the nerves cause a nerve injury-like microenvironment. In this tumor-nerve niche, SCs undergo reprogramming to promote PNI and tumor innervation, eventually constituting positive feedback of the cancer-nerve crosstalk (schematic in Figure 1). SCs can also directly interact with tumor cells and other cellular components in the TME.

## 3. Interactions between SCs and Tumor Cells

Deborde's team demonstrated that through direct physical contact with tumor cells, SCs could interrupt cell-to-cell connections in tumor cell clusters to disperse single cells, which promotes their migration away from the cluster. These activities depend on the expression of neural cell adhesion molecule 1 (NCAM1) on SCs. Simultaneously, the matrix metalloproteins (MMPs) that are secreted by SCs, especially MMP2 and MMP9, enhance the degradation of ECM and provide a trajectory for the movement of cancer cells. As observed through time-lapse images, SCs can also promote the formation of directional protrusions of cancer cells and guide them to neurites. This process is reminiscent of the axon extension induced and guided by SCs during nerve repair [28, 33]. In pancreatic cancer, the SLIT2 expression is lower than that in normal pancreatic tissue. This escape from repellent SLIT2/ROBO signaling helps SCs migrate to cancer cells and contributes to the mutual chemical attraction between cancer and nerves [67]. Sroka et al. reported that the myelinating phenotype of SCs in the TME could regulate the laminin receptor A6B1 and its variant A6pB1 to promote prostate and pancreatic cancer tumor invasion on laminin [68]. In some neurotropic cancer types, such as pancreatic cancer, cervical cancer, and colon cancer, the proliferation of tumor cells can be promoted by SCs in a coculture system, which indicates that paracrine action is also an important mechanism of the crosstalk between SCs and tumor cells [69]. In a recent study by Roger et al., proteomic analyses achieved through mass spectrometry experiments were used to characterize the secretomic characteristics of SCs after coculture with pancreatic cancer cells. The results show that SCs were a source of numerous proteins related to cell movement and cell adhesion [70]. Additionally, in pancreatic cancer, Ferdoushi et al. delineated the secretome of SCs and identified seven secreted proteins that promoted the tumor proliferation or invasion, including MMP-2, cathepsin D, plasminogen activator inhibitor-1, galectin-1, proteoglycan biglycan, galectin-3-binding protein, and tissue inhibitor of metalloproteinase-2 [71]. Other important molecules that mediate interactions between SCs and tumor cells are summarized in Table 1.

## 4. Interactions between SCs and Other Cells in the TME

**Figure 2:**
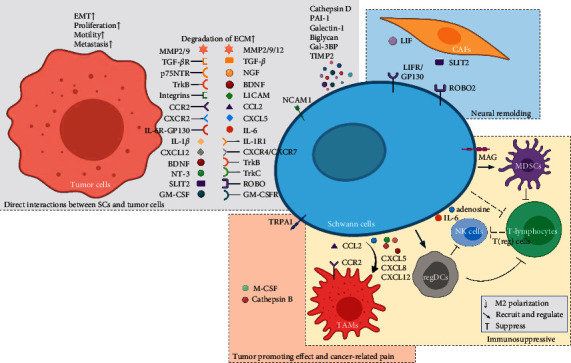
Schematic diagram of how Schwann cells (SCs) interact with tumor cells and other cells in the tumor microenvironment (TME). SCs can affect tumor cells through direct physical contacting and paracrine effects, which promote the EMT, proliferation, motility, and metastasis of cancer. SCs interact with other cells in the TME, involving the formation and maintenance of the immunosuppressive microenvironment, mediating cancer-related pain and tumor neural remodeling. Abbreviation: BDNF, brain-derived neurotrophic factor; CAFs, cancer-associated fibroblasts; CCL, C-C motif chemokine ligand; CCR, C-C motif chemokine receptor; CXCL, C-X-C motif chemokine ligand; CXCR, C-X-C motif chemokine receptor; ECM, extracellular matrix; EMT, epithelial-mesenchymal transition; Gal-3BP, galectin-3-binding protein; GM-CSF, granulocyte-macrophage colony-stimulating factor; GM-CSFR, granulocyte-macrophage colony-stimulating factor receptor; IL-1*β*, interleukin-1*β*; IL-1R1, interleukin-1 receptor type 1; IL-6, interleukin-6; IL-6R, interleukin-6 receptor; LACAM, L1 cell adhesion molecule; LIF, leukemia inhibitory factor; LIFR, leukemia inhibitory factor receptor; MAG, myelin-associated glycoprotein; MDSCs, myeloid-derived suppressor cells; MMP, matrix metalloprotein; NGF, nerve growth factor; NK, natural killer; NT-3, neurotrophin-3; PAI-1, plasminogen activator inhibitor-1; p75NTR, p75 neurotrophin receptor; regDCs, regulatory dendritic cells; SCs, Schwann cells; TAMs, tumor-associated macrophages; T(reg) cells, regulatory T cells; TGF-*β*, transforming growth factor-*β*; TIMP2, tissue inhibitor of metalloproteinase-2; TME, tumor microenvironment; TNF-*α*, tumor necrosis factor-*α*; TrkA/B/C, tropomyosin-related receptor tyrosine kinases A/B/C; and TRPA1, transient receptor potential ankyrin-1.

In addition to tumor cells, SCs can extensively interact with various cells in the TME and participate in some cancer biological behaviors, such as the formation and maintenance of the immunosuppressive microenvironment [30, 32, 52], mediating cancer-related pain [85], and tumor neural remodeling [86, 87] (schematic in Figure 2).

### 4.1. Interactions between SCs and Immune Cells

The reprogrammed SCs can secrete many chemokines and cytokines to recruit and regulate immune cells to form an immunosuppressive microenvironment that is conducive to tumor progression [30, 32, 52]. SCs stimulated by melanoma exhibited the ability to attract and polarize macrophages to the M2 phenotype, and M2 phenotype macrophages could promote tumor progression through various mechanisms, including immunosuppression [32, 88]. SCs can also activate and recruit myeloid-derived suppressor cells (MDSCs) into the TME and enhance their ability to inhibit the proliferation of T lymphocytes. This is achieved by the upregulation of myelin-associated glycoprotein on SCs after exposure to tumor cells [30]. SCs can attract traditional dendritic cells (DCs) and polarize them into regulatory dendritic cells (regDCs) to exhibit powerful immunosuppressive properties [52]. In the newly submitted manuscript by Shurin et al., SCs can directly inhibit the activity of T lymphocytes by polarizing them into a regulated and depleted phenotype in a similar manner to macrophages, MDSCs, and DCs [27]. In lung cancer, C-C motif chemokine ligand (CCL) 2, C-X-C motif chemokine ligand (CXCL) 5, CXCL8, and CXCL12 secreted from SCs decrease the expression of the M1 marker CD80 and upregulate the M2 marker CD206/CD163 in monocytes. M2 phenotype macrophages have immunosuppressive characteristics and can further promote the proliferation of cancer cells [89]. The study by Bakst et al. shows that cancer in the early stage can induce the release of CCL2 from SCs. Through the CCL2/CCR2 axis, inflammatory monocytes are recruited to the site of PNI, where they differentiate into macrophages and secrete more cathepsin B to further destroy the perineurium and enhance tumor invasion of nerves [90]. De Logu et al. illustrated a feedforward mechanism involving macrophages and SCs in mouse models of cancer-related pain. Resident macrophages in the sciatic nerve expanded by their own oxidative burst and targeted SC transient receptor potential ankyrin-1 (TRPA1) activation. TRPA1 on SCs amplified the oxidative burst and released MΦ colony-stimulating factor (M-CSF). M-CSF could further sustain resident macrophage oxidative stress expansion and target neuronal TRPA1 to signal allodynia [85]. Furthermore, it is known that natural killer (NK) cells can independently exert their cytotoxic functions of major histocompatibility complex (MHC)-mediated antigen presentation and thus play an important role in the immunosurveillance of tumors [91]. As illustrated by Lundgren et al., the high infiltration rate of CD56^+^ NK/NK T cells was associated with prolonged survival in patients with periampullary adenocarcinoma [92]. However, in the TME with an immunosuppressive characteristic, there is profound crosstalk between NK cells and a broad cell population. Regulatory T cells (T(reg) cells), M2 phenotype macrophages, and tumor cells themselves can inhibit the activities of NK cells by secreting chemokines, thus promoting tumor immune escape [93]. Adenosine impairs NK cell metabolism by binding to adenosine A_2A_ receptor (A_2A_R) [94]. In addition, there is evidence that IL-6 from the TME of pancreatic cancer can also inhibit the functions of NK cells [95]. Although little has been reported about the direct interaction between SCs and NK cells, SCs in the TME are the key players responsible for the immunosuppressive features and secretion of the above molecules, which might indirectly induce negative effects on the function of NK cells. NK T cells have a role in immune surveillance similar to that of NK cells, but their role in the antitumor response has not been as widely portrayed as that of NK cells [96]. A recent review by Zhang et al. described that in patients with Guillain-Barre syndrome and chronic inflammatory demyelinating polyneuropathy, markers of antigen-presenting cells (APCs), such as CD74, CD1a, CD1b, CD1d, and CD80, were detected on SCs, supporting the presence of their interactions with NK T cells [52]. Since the immune microenvironment in patients with tumor is also unique, further exploring the interactions between SCs and NK T cells in the TME might be interesting. Im et al. found that CD1d was widely expressed on the surface of both primary SCs and immortalized SC lines. In a CD1d-dependent manner, SCs were able to activate NK T cells to produce anti-inflammatory cytokines and suppress the inflammatory response of peripheral nerves. They also proposed that activation of APCs, such as SCs, by agonists could lead to the initiation of anti-inflammatory or immunosuppressive programs via NK T cells [97]. Overall, we still do not fully understand the interactions between NK/NK T cells and the other cellular components in TME, especially SCs. However, given its important tumor immunomodulatory role, especially in the deeper context of the tumor-nerve-immune axis, future studies focusing on this will be essential.

### 4.2. Interactions between SCs and Cancer-Associated Fibroblasts (CAFs)

Cells in the TME, mainly CAFs, can secrete a high amount of SLIT2 and combine with ROBO2 on SCs. The SLIT2/ROBO2 signal transduction further regulates the *β*-catenin or cadherin 2 pathway to enhance the proliferation migration and mitochondrial function of SCs and subsequently promotes PNI and pancreatic ductal adenocarcinoma-associated neural remodeling (PANR) [86]. Leukemia inhibitory factor (LIF) from fibroblasts, macrophages, and mast cells can combine with LIFR and gp130 on SCs to activate JAK/STAT3/Akt intracellular signal transduction. This eventually causes the upregulation of POU3F2, S100, and p21 mRNA levels in SCs and promotes PANR [87]. Li et al. found the role of pancreatic stellate cells (PSCs) in promoting PNI, which involved the activation of the Hedgehog pathway [98]. Therefore, it can be speculated that PSCs may cooperate with SCs, as existing evidence has shown that ephrin-B on fibroblasts in nerve injury can activate the EphB2 receptor on SCs to promote axon genesis [99].

## 5. Conclusions and Future Perspective

Although there have been many attempts to describe the biological behaviors in the tumor-nerve niche, the current models widely used for this area, such as transwell coculture systems [70] and *in vitro* dorsal root ganglion culture in Matrigel [100], have obvious limitations and are not sufficient to fully imitate the interaction of various cell components in cancer-nerve interactions. With the technological advancements of biomaterials and three-dimensional culture platforms (spheres and organoids) [40, 101], we should pay more attention to the multicell crosstalk in the TME, especially the functions of SCs in tumorigenesis and tumor progression. In addition, we have proposed in this review article that SCs undergo reprogramming in the tumor-nerve niche and have profound regulatory effects on immune cells. Based on these inferences, the existing and emerging functions of SCs in two areas were well conducted: regeneration and cancer. It also provides enlightenments for future research on the cancer-nerve-immune axis. Regulating the promoting role of SCs in PNI and tumor innervation and/or blocking their interactions with other cells in the TME may represent new methods to suppress tumor progression. Especially inspired by the combination of antiangiogenesis and immune therapy [102], targeting SCs is expected to be an adjuvant antitumor strategy that combines neurotherapy and immunotherapy.

## Figures and Tables

**Table 1:** Molecules involved in the Schwann cell (SC)-tumor interactions.

Molecules		Mechanisms	Refs
Neurotrophins	NGF	(i) The highly specific and strong affinity chemical attraction between NGF and p75NTR mediated the migration of SCs to pancreatic cancer cells and colon cancer cells instead of normal cells. After using small-molecule inhibitors of TrkA and p75 NTR to block the p75NTR signaling pathway, this chemoattraction process was inhibited.	[36]
	BDNF	(i) SC-released BDNF activated the BDNF/TrkB signaling pathway to promote EMT in salivary adenoid cystic carcinoma, which was represented by the downregulation of E-cadherin and the upregulation of N-cadherin and vimentin, mesenchymal-like morphology changes, and enhanced invasion and migration capabilities.	[72–74]
		(ii) In head and neck squamous cell carcinoma, SCs and tumor cells both highly expressed BDNF and TrkB. The BDNF/TrkB signaling axis is crucial to increase SC migration and tumor metastasis.	
	NT-3	(i) In salivary adenoid cystic carcinoma, tumors secreting NT-3 are bound to TrkC on SCs. Activation of the NT-3/TrkC signaling axis promotes the directional migration and inhibits the cell apoptosis of both SCs and tumor cells. SCs can move to tumor cells before the tumors invade nerves and stimulate tumors to release more NT-3. This phenomenon forms a positive feedback axis to regulate the development of PNI and leads to poor prognosis.	[75]

Cytokines	CCL2	(i) In the TME of cervical cancer, SC upregulates the CCL2 secretion, and tumor cells upregulate the CCR2 receptor. Enhanced CLL2/CCR2 signal transduction promotes the proliferation, migration, invasion, and EMT of cervical cancer and invasion along the sciatic nerve. In turn, tumor cells promote SCs to secrete more MMP-2, MMP-9, and MMP-12, which enhances the degradation of ECM to eliminate tissue obstacles for the movement and migration of SCs and tumor metastasis.	[76]
	CXCL5	(i) In lung cancer, SC-derived CXCL5 is upregulated, and CXCL5 binds to CXCR2 to activate the PI3K/AKT/GSK-3*β*/Snail/Twist signaling pathway in tumor cells, which enhances the invasion and metastasis of lung cancer via EMT.	[77]
	CXCL12	(i) Tumor or hypoxia induces the high expression of CXCR4/CXCR7 in SCs, which is recruited by CXCL12 from pancreatic cancer cells; thus, it initiates the cancer-nerve contact in the early stage of tumors. This CXCL12-dependent mechanism can also suppress intrinsic molecular pain pathways and spinal astrocytes and microglia in SCs *in vivo* to decrease the pain sensation.	[78]
	GM-CSF	(i) In pancreatic cancer, the expression of HIF-1*α* is upregulated and induced the secretion of GM-CSF by tumor cells. GM-CSF can promote the migration of SCs, which promotes the tumor-nerve interactions and occurrence of PNI.	[79]
	IL-6	(i) In the interactions between pancreatic cancer and SCs, IL-1*β* secreted by tumor cells combines with IL-1R1 on SCs to activate the nuclear factor (NF)-*κ*B pathway, which increases the production of cytokines, including IL-6, in SCs. In turn, IL-6 activates the STAT3 signaling pathway in cancer cells by binding to the IL-6R-GP130 complex to promote EMT, migration, and invasion of cancer cells.	[80, 81]
		(ii) Hypoxia and IL-6 secreted from tumor cells induce the reactive gliosis of SCs that suppress spinal astroglia and microglia; thus, they inhibit the painful conduction in the early stages of tumors.	
	TNF-*α*	(i) TNF-*α* is overexpressed in oral cancer and can activate and recruit SCs. The activated SCs increase the proliferation and migration and release more TNF*α* and NGF to promote cancer proliferation, progression, and nociception.	[82]
	TGF-*β*	(i) A large amount of TGF-*β* secreted by SCs activated the TGF-*β*/SMAD signaling pathway in pancreatic cancer cells to induce EMT to promote the migration, invasion process, and PNI.	[70]

Others	Adenosine	(i) The interactions between SCs and oral squamous carcinoma cells increase adenosine production, which stimulates the cell proliferation and migration of two cell types through binding to ADORA2B and further stimulates the secretion of IL-6 in SCs.	[83]
	L1CAM	(i) In pancreatic cancer, SCs secrete soluble L1CAM combined with integrin on tumor cells and activate the STAT3 kinase signaling pathway to increase the secretion of MMP-2 and MMP-9 and promote PNI.	[84]

Abbreviation: A6BA, laminin-binding integrin A6B1; ADORA2B, adenosine receptor A2B; BDNF, brain-derived neurotrophic factor; CCL, C-C motif chemokine ligand; CCR, C-C motif chemokine receptor; CXCL, C-X-C motif chemokine ligand; CXCR, C-X-C motif chemokine receptor; ECM, extracellular matrix; EMT, epithelial-mesenchymal transition; GM-CSF, granulocyte-macrophage colony-stimulating factor; HIF-1*α*, hypoxia-inducible factor-1*α*; IL-1*β*, interleukin-1*β*; IL-1R1, interleukin-1 receptor type 1; IL-6, interleukin-6; IL-6R, interleukin-6 receptor; LACAM, L1 cell adhesion molecule; MMP, matrix metalloprotein; NF-*κ*B, nuclear factor-*κ*B; NGF, nerve growth factor; NT-3, neurotrophin-3; p75NTR, p75 neurotrophin receptor; PNI, perineural invasion; SCs, Schwann cells; STAT3, signal transducer and activator of transcription 3; TGF-*β*, transforming growth factor-*β*; TME, tumor microenvironment; TNF-*α*, tumor necrosis factor-*α*; TrkA/B/C, tropomyosin-related receptor tyrosine kinases A/B/C.

## Data Availability

This is a review article, and therefore, the data discussed in this study are publicly available.
